# The role of video and reflection interventions in supporting first-year medical students in the gross anatomy lab: a mixed methods study

**DOI:** 10.1186/s12909-026-08673-2

**Published:** 2026-02-26

**Authors:** Emily L. Bradshaw, Katherine Daly, Heather Rashal, Xiang Zhu, Jeffrey H. Plochocki

**Affiliations:** 1 Department of Medical Education, College of Medicine, University of Central Florida, 4850 Lake Nona Blvd, Orlando, FL 32827 USA; 2 Department of Clinical Science, College of Medicine, University of Central Florida, Orlando, FL USA; 3 Burnett School of Biomedical Sciences, College of Medicine, University of Central Florida, Orlando, FL USA

**Keywords:** Gross anatomy education, Dissection, Anxiety, Video, Reflection

## Abstract

The first encounter with a human cadaver is a sentinel experience for medical students that is an important professional milestone but can be associated with heightened stress and anxiety. In response, many anatomy programs have implemented educational and wellness-based interventions to prepare learners for their first dissection experience, manage their dissection-related anxiety, and foster empathy. This mixed methodology study examined the impact of two preparatory interventions, namely a video and reflection activity, on anxiety, sleep, and mental and physical health for fifty-eight first-year medical students. As expected, anxiety increased over the anatomy module. There were no observed changes to sleep during the module. Self-reported number of physically and mentally healthy days improved over the module. Qualitative responses indicate that the students valued the video and reflection activity and found them helpful in preparing them for and adapting to the anatomy experience, and that male participants found them more helpful than female participants. Engagement in specific health promotion, namely exercise and maintaining a strong social support system, seemed to be protective against stress and anxiety. More research is needed to determine the benefits of preliminary activities to support healthy adjustment and reduced anxiety for students taking anatomy.

## Background

The dissection of donated human remains is a cornerstone of anatomy instruction in undergraduate medical institutions that enhances learning outcomes and fosters a psychosocial understanding of death and dying [14, 18, 33]. However, for many medical students, human dissection represents a novel experience, and without adequate preparation, their initial encounter with a human donor can be emotionally traumatic [12]. While the stress associated with dissection reportedly diminishes over the course of an anatomy program [27], it can lead to desensitization towards death and the development of emotional distancing behaviors and attitudes towards patients [16, 21]. A year later, students often retain vivid memories of their first dissection experience and report developing ambivalent feelings towards the human body [19].

In response, many anatomy programs have implemented educational and wellness-based interventions to prepare learners for their first dissection experience, manage their dissection-related anxiety, and foster empathy. One common approach involves showing learners a video before their first dissection. These videos aim to humanize the donors, increase student empathy, and alleviate anxiety [5]. They expose students to the anatomy laboratory environment and provide information about the body donation process to mitigate apprehension [7]. However, studies have shown that such video interventions have limited effectiveness in reducing student stress [4, 17] and may even exacerbate anxiety towards dissection [3, 13]. One study associated video preparation with an increase in physiological stress indicators, such as fainting, nausea, appetite loss, and sleep disturbances [3].

Reflection exercises represent another approach that may help students emotionally prepare for and process the dissection experience [11]. These exercises can help normalize the emotions students feel prior to dissection, cultivate humanism and compassion, and promote psychological wellness [1, 36, 37]. Many medical students find it beneficial to discuss the emotional aspects of the first donor encounter with other students and an anatomist or clinician as a coping strategy [8, 32]. Thus, exercises that incorporate personal and cooperative reflections with peers and instructors on their first dissection may prove beneficial. However, for reflection exercises to be effective, they require a meaningful prompt that allows learners to reflect in a deep and critical way [2]. Therefore, a reflection exercise facilitated by an anatomist or physician, following the viewing of a dissection video, may improve attitudes towards dissection and reduce anxiety.

This study investigates the impact of pre-dissection video-based learning and guided reflection exercises on reducing anxiety among medical students. Additionally, it evaluates how demographic factors, prior anatomical exposure, and individual coping mechanisms influence anxiety levels. We hypothesize that the combined use of these interventions will mitigate anxiety across both pre- and post-dissection phases during students’ first cadaveric experience.

## Methods

### Study design and participants

This pre-post intervention study assessed the effects of pre-anatomy lab activities on anxiety levels among first-year medical students (M1s) at the University of Central Florida College of Medicine during Fall 2023. The Institutional Review Board at the University of Central Florida reviewed the protocol (IRB# STUDY00005744) and granted exempt status.

All 120 enrolled M1 students received study information via email in August 2023, followed by three reminder emails. Eligibility criteria included: (a) age ≥ 18 years and (b) current enrollment as a first-year medical student at UCF. Participation was voluntary, and all students were enrolled in the *Structure and Function* module—a 20-week preclinical course covering anatomy and physiology that included 32 h of anatomy laboratory instruction involving human donor dissection.

## Materials

### Videos

The M1 curriculum required students to watch two instructional videos: the short biographical feature *Virginia Herald Story* [13] and the documentary *Anatomy and Humanity* [10]. These resources were selected for their relevance to medical education, emphasizing both anatomy instruction and the human dimension of cadaver donation. Specifically, they provided detailed insights into donor experiences and the ethical rationale behind the donation process, as well as medical students’ perspectives and emotional responses to dissection. All students were instructed to watch Video 1 prior to the session; 115/120 (96%) of students watched Video 1 in its entirety. The other 5/120 (4%) students watched approximately half of video 1. During the session, all students watched Video 2 in its entirety.

*Video 1: Virginia Herrald Story* – This 13-minute documentary describes the life of Virginia Herrald and her decision to donate her body for anatomy education. This video was produced as a part of the “Willed Body Donor Oral History Project” at the UCF College of Medicine.

*Video 2: Anatomy and Humanity* – This 15–minute documentary describes the stories of anatomical body donors and interviews with medical students about their personal experiences with dissection. The video also includes an anatomy professor’s interaction with a donor (UCF College of Medicine emeritus anatomy faculty).

### Survey instruments

M1 students were invited to complete six self-report surveys through Qualtrics (Qualtrics, Provo, Utah) during the Structure and Function module. This was a repeated measures collection. Each survey contained one or more of the following measures: baseline measures, State-Trait Anxiety Inventory **(**STAI), Centers for Disease Control and Prevention (CDC) Healthy Days, Patient-Reported Outcomes Measurement Information System (PROMIS) Sleep Disturbance, and intervention effectiveness and open-ended questions. Survey measures are available in supplemental materials (see SA1). The surveys were quasi-voluntary, as participants may have felt pressure to complete the surveys. Before the study began, surveys were previewed by two authors to examine the layout. After all surveys were submitted, one author verified there were no duplicated surveys.

#### Baseline measures

M1 students were invited to complete fifteen categorical, demographic items including ethnicity, age, sex, previous anatomy course and human dissection experience, and time engaged in coping/support behaviors each week. Coping behavior options were provided to participants as a list which included exercise/physical activity, meditation/mindfulness, time in nature, time with a pet, faith/spirituality, expressive arts, journaling, social support, and as well as an open response option.

#### State-Trait Anxiety Inventory (STAI)

The STAI is a well-established scale composed of two parts, which measure state and trait anxiety, and is reported to have good validity; Cronbach’s alpha reported as 0.91 [30]; Mind Garden, Menlo Park, CA). We used the value of 0.70 or higher as being acceptable [24]. In this study, we utilized the STAI-6 which includes six items focused on how the participant feels at that moment rated on a four-point Likert-scale (range: “not at all” to “very much”); Cronbach’s alpha is reported as 0.82 [20]. Measures were scored according to the standard instructions. Higher scores indicate higher anxiety.

#### PROMIS sleep disturbance (Short Form 8a)

The Patient-Reported Outcomes Measurement Information System (PROMIS) asks participants to self-report their perceptions of sleep disturbances and sleep-related impairment during the past seven days on a Likert scale. In this study, we used the sleep disturbance short form which contains eight items rated on a five-point Likert-scale (range: not at all to very much, or never to always). It has good validity and reliability; Cronbach’s alpha is reported as 0.91 [28]. Data were scored according to the standard instructions and scores were summed to yield a global score [35]. Higher scores indicate more sleep disturbance.

#### CDC healthy days survey

This survey evaluates health-related quality of life (CDC HRQOL-4) measures. It includes four items: (a) self-rated general health using a five-point Likert-scale (range: poor to excellent) as well as free responses to the total number of days in the last month when a person was (b) physically unhealthy, (c) mentally unhealthy or (d) limited in usual activities due to poor physical or mental health [22]. Cronbach’s alpha is reported as 0.76 [31, 34]. Higher scores on the self-rated health question indicate better health.

#### Helpfulness of pre-laboratory activity survey

Participants were asked to complete three items about the helpfulness of (a) Video 1, (b) Video 2, and (c) the reflection session using a Likert-type scale (1–4: Not at all helpful to Extremely helpful). Higher scores indicate the activity is rated more helpful.

#### Qualitative questions

Participants were asked to respond to five free response items about their perception of whether their experience in the anatomy laboratory impacted their mental health, physical health, or sleep. Additionally, participants were asked if there were characteristics specific to their donor or laboratory student group that impacted their lab experience.

### Procedure

**Fig. 1:**
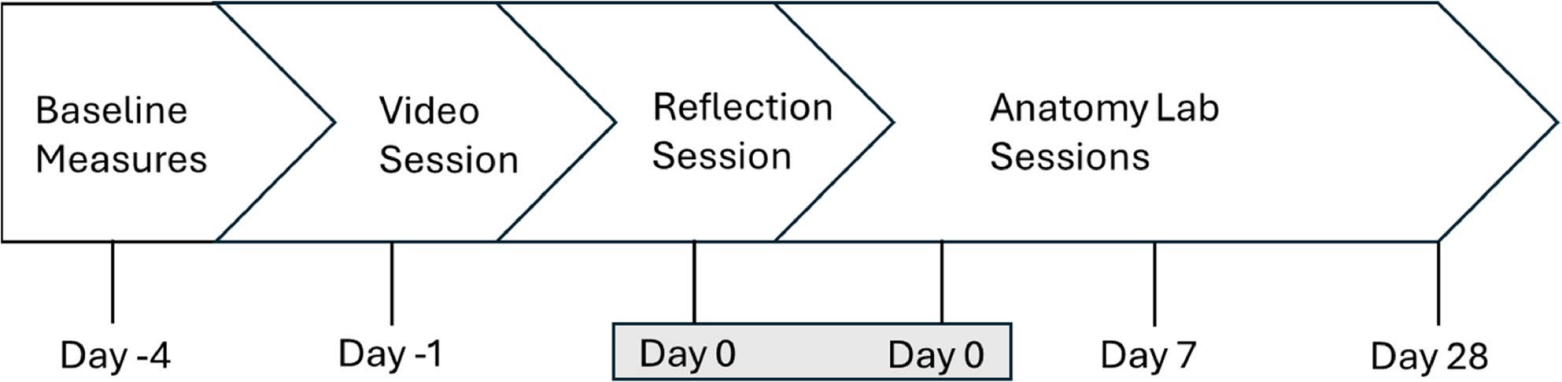
Study timeline. The timeline corresponds with the anatomy laboratory start date. Baseline measures were collected from day − 5 to day − 4 and consisted of demographic data, anatomy and dissection experience, coping behavior use and frequency, STAI, PROMIS, and CDC Healthy Days measures. After the video, reflection and first anatomy lab sessions, students were invited to take the STAI. After anatomy labs 2 and 4 (day 7 and 28), STAI, PROMIS, CDC Healthy Days, and pre-lab intervention helpfulness questions were included on the survey

Study enrollment began in August 2023 during the 2023–2024 academic year. Figure 1 shows the overview of the procedure timeline; day 0 corresponds to the reflection session and the first anatomy laboratory. Students who completed the baseline survey consisting of thirty-four items received a $10 Amazon gift card incentive.

#### Pre-Anatomy laboratory video intervention

Prior to the first anatomy laboratory (day − 1), all M1 students were required to watch a documentary video (Video 1) and were then invited to complete the STAI survey.

#### Pre-anatomy laboratory reflection session intervention

Before the first anatomy laboratory (day 0), all students participated in a reflection session. Groups of twelve students met with a faculty member or teaching assistant (TA) from the anatomy laboratory and watched a documentary video (Video 2). Students were given twenty minutes to reflect on both videos and to provide handwritten answers to two questions: (a) How do you think the videos impacted your views towards the anatomy laboratory experience? (b) How do you anticipate reacting to handling and dissecting donors in the anatomy laboratory? The students were told not to include their names to maintain anonymity. Faculty members, TAs, or students read the written responses. Student groups discussed their attitudes, concerns, and emotions concerning dissection, anatomy laboratory experience, and coping mechanisms. After the reflection session, students were invited to take the STAI survey.

#### First anatomy laboratory session

Immediately following the reflection session, all M1 students participated in their first anatomy laboratory. Tasks included physical examination of the donor’s body and evaluating the integumentary system. This evaluation included palpation and making the first cut to remove skin on the back. The duration of the laboratory was two hours. After the first anatomy laboratory, students were invited to take the STAI survey.

#### Subsequent anatomy laboratory sessions

Students met once each week for a four-hour anatomy laboratory session. After the second lab session (day 7) and the fourth lab session (day 28), M1 students were invited to complete surveys. After the fourth laboratory, participants who completed at least four out of five surveys (video, reflection, lab 1, post-7 and post 28) received a $10 Amazon gift card incentive.

### Statistical analysis

Statistical analysis was performed using Stata MP v.15.1 (StataCorp LLC). Descriptive statistics were estimated using mean ± SD for continuous variables and percentages for categorical variables to show demographic characteristics of the study population. Multiple regression analysis was conducted to evaluate the effect of intervention of video I, video II and reflection at different time points on participants’ stress score, perceived helpfulness of the interventions, and stress-related sleep quality, general health, physical health, and mental health. In this analysis, we first checked the distribution nature of each dependent variable using Shapiro-Wilk test and histogram graphs. If a dependent variable exhibited a normal distribution, a multiple panel linear regression model was applied, otherwise, a multiple nonlinear panel (either negative binomial or ordinal logit) regression model was used depending on its distribution and scale nature. In these analyses we assumed that there was no time-associated effect in the short study period, and that all detected effects if any are due to the intervention and gender with both these factors defined as fixed effect and individual ID as a random effect in the analysis. Non-parametric rank sum test was used to compare the difference between female and male, and between low STAI and high STAI participants, on the frequency of each stress coping activity taken. All tests were two-tailed with α < 0.05 considered significant.

## Results

Ninety students completed at least two surveys and were included in this study (90/120, 75% absolute response rate). Of the 90 students, 55 of them completed the baseline measures (61% relative response rate; see Table 1). The sex distribution in the respondents was 53% male and 47% female (*n* = 31 and *n* = 27, respectively). Just over half of the participants (52%) had taken an anatomy course, and only two reported dissecting a human donor (4%, data not shown).

**Table 1:** Demographic characteristics of participants. Fifty-eight participants responded to the baseline demographic survey 64.4% (58/90)

Item	*N*	%	Total
Ethnicity			
Hispanic/Latino/Latinx	4	6.9	6.9
African American/Black	3	5.17	12.07
Asian/Pacific Islander	23	39.66	51.72
White/non-Hispanic	26	44.83	96.55
Other	2	3.45	100
Sex			
Male	31	53.45	53.45
Female	27	46.55	100
Previous anatomy course			
Yes	33	57.89	57.89
No	24	42.11	100
Age	Mean	SD	Range
	23.55	2.8	21–39

### Student sleep

**Fig. 2:**
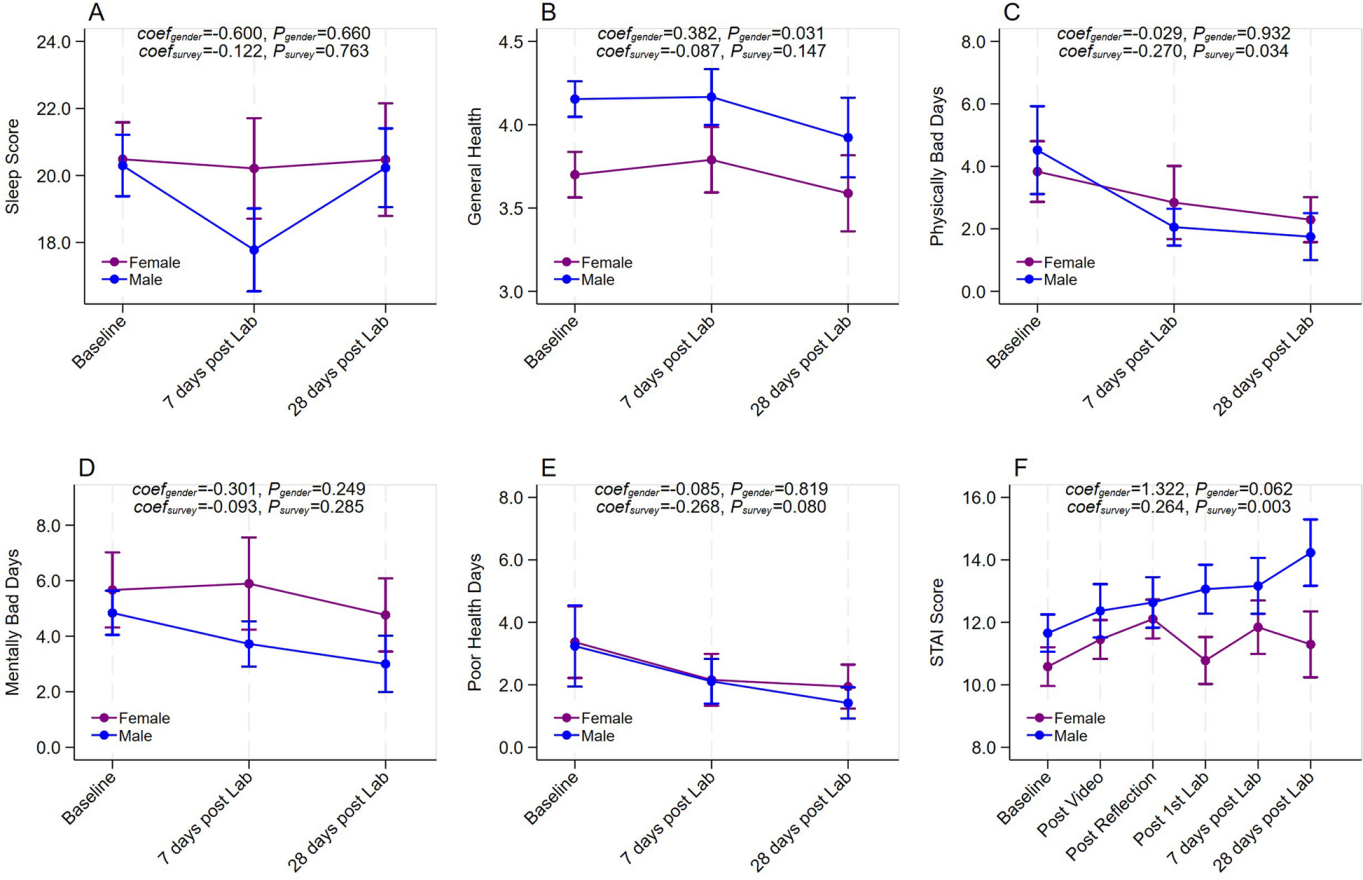
Sleep score (**A**), general health score (**B**), number of physically bad days (**C**), number of mental bad days (**D**), number of poor health days (**E**), and overall STAI score, varied over time in response to the intervention of video, reflection, and lab work. Participants who completed at least two times of surveys are included. Multiple panel linear regression model was applied in (**A**), (**B**) and (**F**). Multiple panel negative binomial regression model was applied in (**C**), (**D**) and (**E**). **A** Response rates for PROMIS are 64% (*n* = 58/90) at baseline and 33% (*n* = 30/90) on day 28. **B** Response rates for CDC Healthy Days self-rated health at baseline are 56% (*n* = 50/90) and 30% (*n* = 30/90) by day 28. **C**, **D**, **E** Relative response rates for CDC Healthy poor health days at baseline are 61% (*n* = 55/90) and 32% (*n* = 29/90) on day 28. **F** Relative response rates for STAI are 63% (*n* = 57/90) at baseline and 33% (*n* = 30/90) by day 28

To determine if there were changes to participants’ sleep as they adjusted to the gross anatomy laboratory experience, we examined PROMIS sleep survey responses. We did not detect any significant changes over time in sleep score (*p* = 0.763, see Fig. 2A).

### Student health

To determine if there were any self-reported changes to participant physical and mental health as they adjusted to the anatomy laboratory, we examined participant responses to the CDC Healthy Days survey. We did not detect any significant changes over time to general health score (*p* = 0.147), number of mentally bad days (*p* = 0.285), or number of poor health days (*p* = 0.080, see Fig. 2B, D, E). However, there was a reduction in physically bad days (*p* = 0.034, see 2 C). Similarly, female participants reported worse general health compared to males (*p* = 0.031, see Figs. 2B and 56% response rate at baseline and 33% at day 28).

### Student state anxiety

To determine if pre-laboratory interventions reduced participant state anxiety, we evaluated the change in STAI score over time in response to the intervention. The STAI score showed a significant increase from the baseline (male: 11.65 ± 3.05; female: 10.58 ± 3.46; 63% response rate) to 28th day post lab (male: 14.23 ± 3.83; female: 11.29 ± 4.35; *p* = 0.003; 33% response rate; see Fig. 2F).

To determine if previous anatomy experience was associated with a change in anxiety, we evaluated STAI scores at baseline and on subsequent surveys. No significant difference was seen (data not shown).

### Perceived helpfulness of the pre-anatomy laboratory interventions

**Fig. 3:**
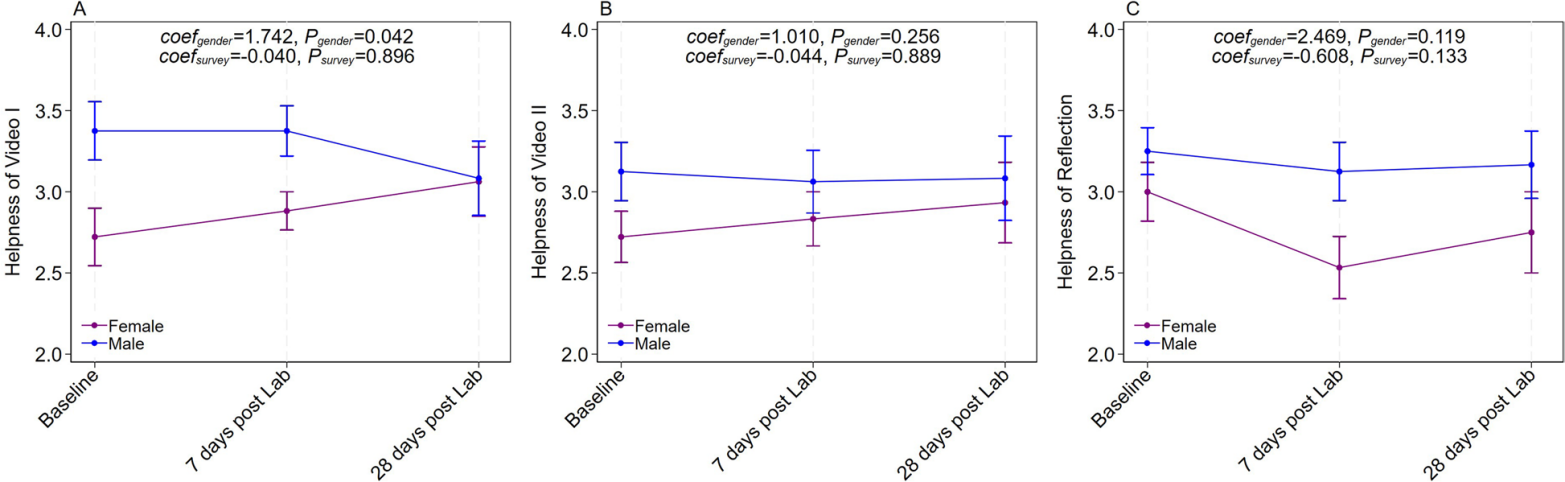
Figure 3. Graphs showing that perceived helpfulness of video I (A), video II (B), and reflection (C). Participants who completed at least two times of surveys are included. Multiple panel ordinal logit regression analysis was performed. The response rate is 38% (n=34) at post-first lab, 37% (n=33) at 7 days, and 26% (n=24) at day 28

Participants were asked to rate the helpfulness of video I, video II, and the reflection session at multiple time points. Both sexes had a relatively consistent feeling post 1st lab, 7 days post lab, and 28 days post lab (*p* = 0.931, *p* = 0.855, and *p* = 0.11; response rates 38% post 1st lab, 37% at 7 days, and 30% at 28 days; see Fig. 3). Males tended to report that the interventions were more helpful than did females, especially on video I (*p* = 0.029).

Spearman’s correlation analysis revealed that STAI score was positively correlated with the helpfulness of video 2 (ρ = 0.319, *p* = 0.048, see Table 2). The helpfulness of video I, video II and reflection were all significantly positively correlated with one another.

**Table 2:** Summary of pairwise correlation analysis among STAI score, perceived helpfulness of video I, video II and reflection at post first lab, 7 days post lab, and 28 days post lab. The values represent spearman correlation coefficient (ρ). * denotes *p* < 0.05. The response rate is 46% (41/90) at post-first lab and 28% (25/90) at day 28

	STAI Score	Helpfulness of video 1	Helpfulness of video 2
Post first lab			
Helpfulness of video 1	0.187		
Helpfulness of video 2	0.013	0.695*	
Helpfulness of reflection	0.092	0.469*	0.305
7 days post lab			
Helpfulness of video 1	0.295		
Helpfulness of video 2	0.167	0.381*	
Helpfulness of reflection	0.128	0.599*	0.408*
28 days post lab			
Helpfulness of video 1	0.150		
Helpfulness of video 2	0.099	0.822*	
Helpfulness of reflection	0.055	0.478*	0.662*

### Student coping strategies

**Fig. 4:**
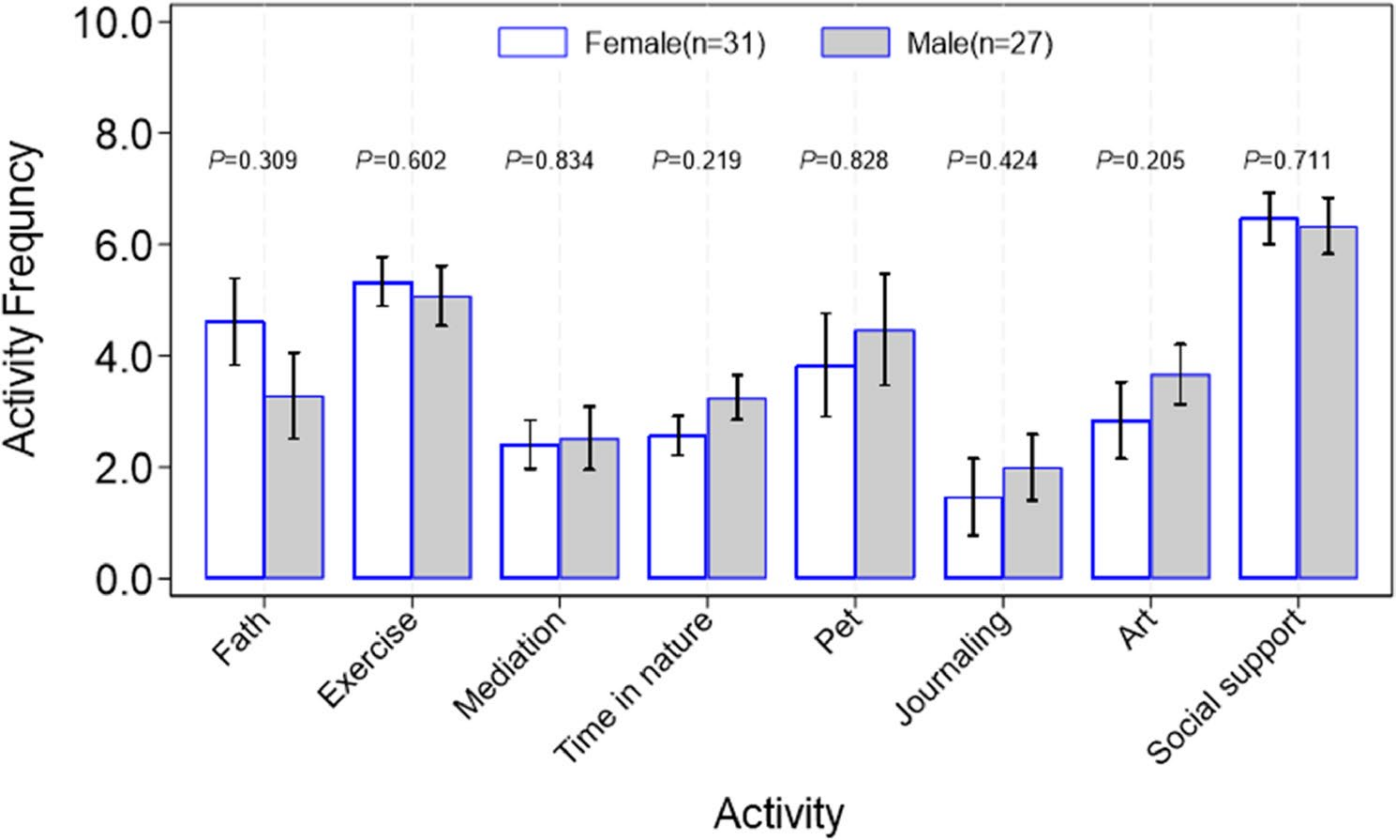
Comparison between female and male on stress coping strategies, and overall distribution of different coping strategies. Non-parametric rank sum test was used to test the difference between female and male on the frequency of each stress coping activity taken. The relative response rate was 34.4% (31/90) for females and 30% (27/90) for males

Medical students may deal with many types of stressors in medical school, and some studies have reported gender differences in coping strategy utilization. In our study, males and females both reported a similar level of coping strategy utilization with social support and exercise being the most frequently used (see Fig. 4).

**Fig. 5:**
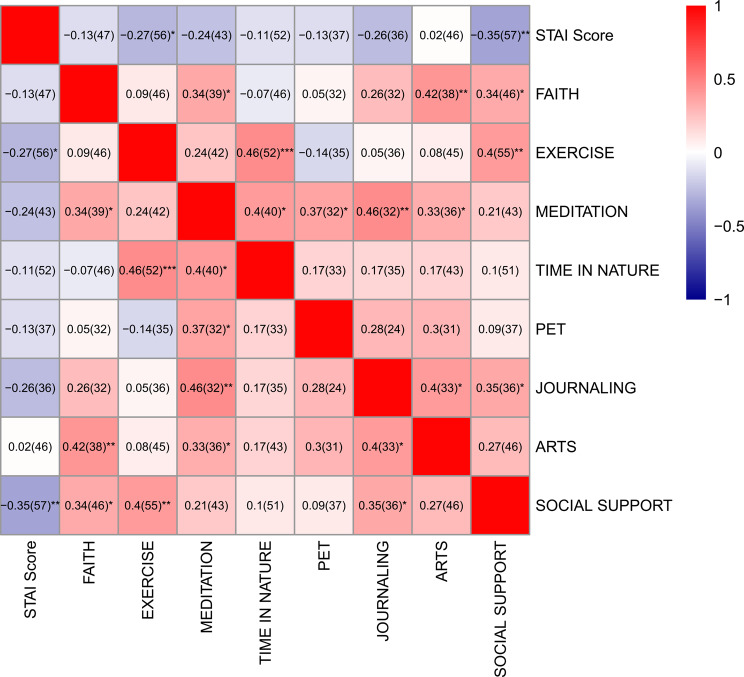
Heatmap showing the correlation between stress coping methods and average STAI score of 6 surveys. Spearman correlation coefficients (ρ) were reported with (n) representing the number of respondents. Spearman correlation coefficients were reported. ***, ** and * denote p-value < 0.001, ≥ 0.001 -< 0.01, and ≥ 0.01- < 0.05 respectively. The relative response rate varied for each coping method; lowest rate was 40% (36/90) and highest was 61% (55/90)

To determine if there was any correlation between student anxiety score and coping strategies, we examined the type and of participant coping strategies and average STAI score. Seven of the eight coping methods were negatively correlated with STAI score, with social support (ρ =-0.35) and exercise (ρ =-0.27) showing the most robust negative correlation (see Fig. 5). The heatmap also shows positive correlations between other forms of coping methods including time in nature and exercise (ρ = 0.46), meditation and journaling (ρ = 0.46), arts and faith (ρ = 0.42), as well as exercise and social support (ρ = 0.4).

**Fig. 6:**
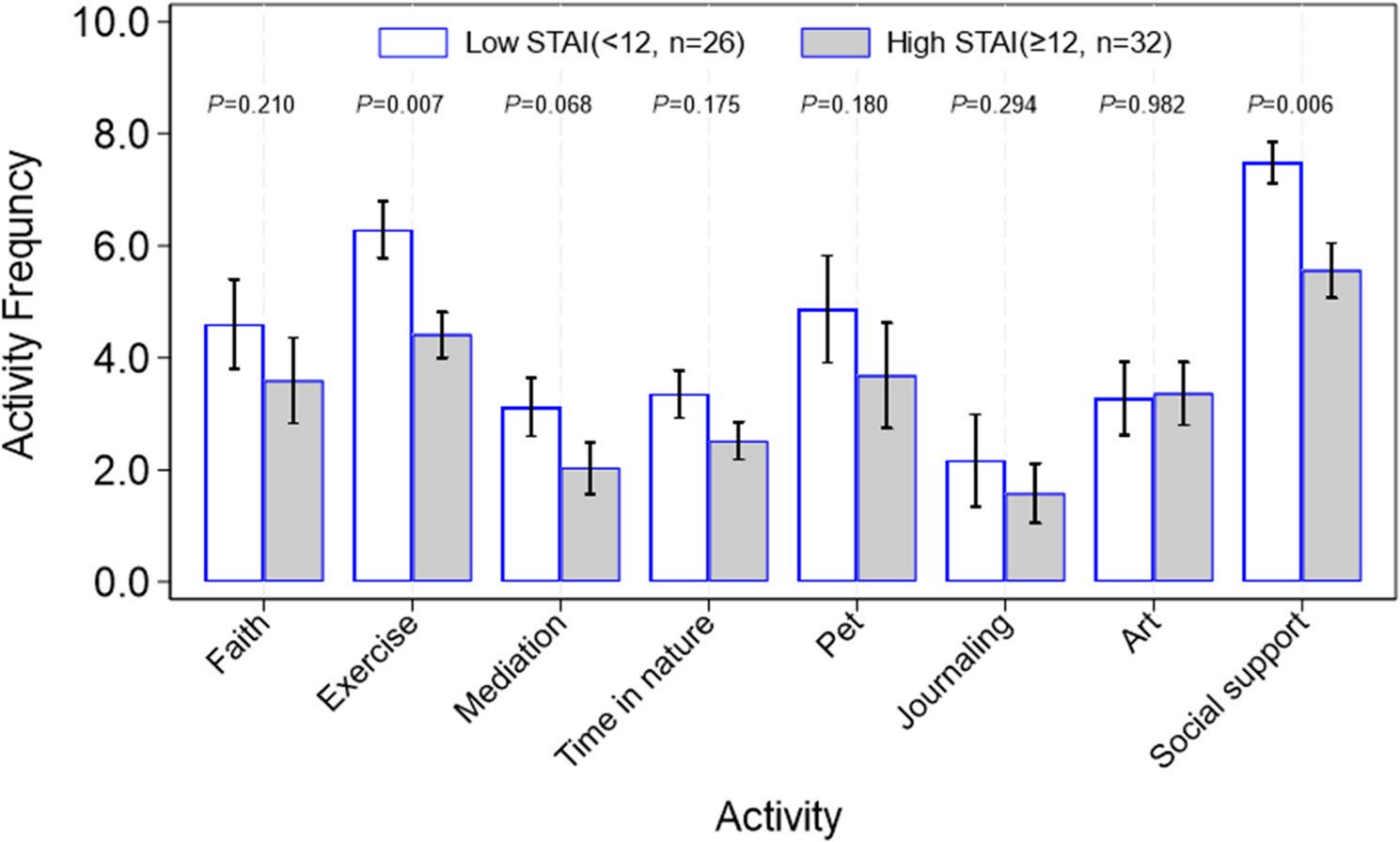
Comparison between participants experiencing low STAI defined as STAI score < 12 and participants with high STAI defined as STAI score ≥ 12 on stress coping strategies, and overall distribution of different coping strategies. Non-parametric rank sum test was used to test the difference between the two categories of participants on the frequency of each stress coping activity taken. The relative response rate was 28.9% (26/90) for low STAI participants and 35.6% (32/90) for high STAI participants

We then separated those who reported low versus high STAI score and examined coping strategy utilization. Low STAI scores tended to be correlated with higher utilization of almost all stress coping methods with exercise and social support being the most significant coping methods (*p* = 0.007 and *p* = 0.006 respectively; see Fig. 6).

### Student perceptions of pre-lab interventions and anatomy laboratories

Participants provided feedback on the pre-lab video and reflection activity interventions through open-ended survey questions. Though a formal thematic analysis was not performed, a total of 116 student quotes are as follows (Table 3). Student responses to the pre-lab interventions show emotional engagement, with many emphasizing the importance of acknowledging the humanity of the donors. Students also expressed gratitude toward the donors and appreciation for the emotional preparation provided before the session.

**Table 3:** Student responses to open-ended survey items. Open-ended survey questions and representative student quotes are shown. One hundred sixteen responses were collected; the response rate for each question is indicated

Survey Item	Representative Student Quotes
*Documentary Videos*: Is there anything else you’d like to share about this experience? Response rate = 16% (19/116)	“The video made me cry. When you learn about the story behind the body, it gives you so much more feelings. But out of all, the gratitude is the most significant.” “I really enjoyed the “Anatomy and Humanity” video. It was humbling to hear about the real stories of some of those individuals that became donors…”
*Reflection Activity*: Is there anything else you’d like to share about this experience? Response rate = 12% (14/116)	“The reflection helped put me at ease and it helped prepare me emotionally for anatomy lab.” “It was good to reflect with classmates and hear different viewpoints and express gratitude to those who donated their body.”
*First Lab*: Is there anything else you’d like to share about this experience? Response rate = 3% (3/116)	““I’m still processing how I feel about that and myself overall… I’m still a bit anxious from navigating that experience while balancing my emotion.” “I am deeply grateful to have this opportunity to learn… It was nice to have watched the humanity videos to better understand how intentional every aspect of the lab is, especially the choice of the individuals to be donors.”
Second laboratory (day 7): Is there anything else you would like to share about this experience. Response rate = 8% (9/116)	“Reflection session…didn’t need to go so long.” “I think it would’ve been better if the submission were online rather than handwritten. Though anonymous, you could tell whose paper it was…” “The reflection session was too close to the lab session…caused me to be a little more cautious and nervous.”
Second Laboratory: In what ways, if any, have your experiences in the anatomy course thus far impacted your *mental* health? Response rate = 28% (32/116)	“Realizing the breadth of anatomy we have to learn in such a short amount of time has increased my general anxiety.” “I feel a bit more stressed due to the amount of anatomical knowledge that I don’t know yet and some self-imposed pressure to perform successful, clean dissections in a limited amount of time.”
Second laboratory: What, if any, characteristics specific to *your cadaver* impacted your experience? Relative response rate = 16% (18/116)	“Our cadaver is a male, with a similar build/physique to my dad and about the same age he was when he died.” “I feel a bit more stressed due to the amount of anatomical knowledge that I don’t know yet and some self-imposed pressure to perform successful, clean dissections in a limited amount of time.”
Second Laboratory: What, if any, characteristics specific to *your lab group* impacted your experience? Relative response rate = 18% (21/116)	“My group is constantly asking each other questions, and this has positively impacted my experience. I do not feel embarrassed when I do not know something, since we are all learning together.” “The experience has been successful partly due to how well my group works together. I think we are all very supportive of each other, we help share information and divide up the work evenly.”

Following the second laboratory session, some students raised concerns about the reflection activity, particularly regarding anonymity and scheduling, though no criticisms were reported for the video intervention. Regarding mental health, comments indicated heightened anxiety stemming from the volume of material to learn and the complex emotions associated with dissection (Table 3). Positive feedback centered on constructive lab group dynamics, including students’ appreciation for diverse backgrounds and collaborative teamwork. These student quotes underscored the value of peer support in navigating both technical and emotional challenges during the sessions.

## Discussion

This study examined psychological variables and changes associated with adapting to the gross anatomy lab experience for first-year medical students. Consistent with the previous literature, the anatomy experience is a sentinel event in the medical student curriculum that can impact students’ wellbeing via increased stress [1, 25]. Albeit it is also marked by interest and excitement for learning [23]. We found that medical students’ anxiety increased over the anatomy module. This corresponds with medical education literature that shows that stress and anxiety symptoms increase during the pre-clinical years of medical school [29] and are particularly heightened around the dissection experience [1].

The interventions studied were reported as beneficial by students of both sexes, but more so by male participants over time. This is an interesting finding. Sex differences in medical students have been studied regarding empathy and rates of burnout [9] with females having higher empathy and higher rates of burnout. Not much has been studied in anatomy literature, but human dissection and narrative writing around the dissection experience has been shown to have psychological benefits in previous qualitative studies (e.g., Abrams et al., [1]. One explanation for the sex difference observed in this study is that the video and experience of reflective writing may have helped men process the complex emotions associated with human dissection, giving them an outlet that they may not have otherwise had.

Students in our study reported a broad repertoire of coping mechanisms used. It appears exercise and social support were two coping strategies negatively correlated with STAI scores, meaning that greater use of the methods was associated with lower anxiety. Other studies have documented that exercise as an effective strategy for reducing anxiety and burnout for pre-clinical medical students [6]. Social support has robust research literature documenting its benefits in reducing stress, anxiety, and many other psychological risk factors for medical students [15]. It is possible that the pre-lab activities in this study offered some degree of social support since they were done in a small group setting where students had the opportunity to interact and process their reactions together.

Sleep is a psychological variable often studied with medical students. Many medical students report sleep disturbances including poor sleep, not sleeping the recommended hours, and insomnia associated with the common mental disorders [26]. In our study we hypothesized that the stress and anxiety of anatomy experience might further exacerbate these common sleep concerns. However, our findings did not support this hypothesis, and no significant sleep issues were identified.

### Limitation

This study had several limitations including small sample size and a higher rate of survey response attrition resulting in limited complete data. Some of this was due to communication errors during the study’s execution. For example, many of the study emails were sent during a transition time between the previous module final exam and the start of the structure and function anatomy module with some students missing the baseline data timeframe but completing subsequent surveys. There was also a likelihood of survey fatigue with numerous survey distributions. 

### Future research

Future research in this area may want to have a true control group where a portion of the class is randomly assigned to participate in one or both interventions with the remaining students receiving no intervention (a true control group). This was not permitted in our study design as there were curricular guidelines ensuring that all students received the same preparation for anatomy (since our wellness interventions were built into the curriculum). Sleep and other aspects of physical health did not yield significant results, but there may be other variables of interest worth examining, such as empathy, engagement in healthy behaviors, professional identify formation, and awareness of one’s own mortality. Many students regard the donor as their “first patient” [1] and identifying interventions that enable them to gain the most from this valuable learning experience both academically and in terms of their professional identity are important. Lastly, a future direction for research on this topic could be a multi-medical school study comparing those with similar anatomy approaches who do not incorporate wellness interventions to those who do on target outcomes (i.e., anxiety, sleep, burnout, satisfaction with learning, etc.)

## Conclusions

This study examined the effects of pre-anatomy laboratory activities on first-year medical student anxiety, health, and sleep as they adapted to the gross anatomy laboratory. We found that medical students’ anxiety increased over the anatomy module, but there were no substantial changes to sleep or self-reported health. Participants rated video and reflection interventions as helpful with male students rating these higher compared to female students. Participants who report higher use of exercise and social support coping strategies also are associated with a lower STAI score. More research is needed on pre-laboratory interventions to reduce medical student anxiety and to enhance wellbeing during this transformational learning milestone.

## Data Availability

The datasets used and/or analyzed during the current study are available from the corresponding author on reasonable request.
